# Relative versus absolute RNA quantification: a comparative analysis based on the example of endothelial expression of vasoactive receptors

**DOI:** 10.1186/s12575-021-00144-w

**Published:** 2021-02-14

**Authors:** Kevin Kuhlmann, Melanie Cieselski, Julia Schumann

**Affiliations:** University Clinic and Outpatient Clinic for Anesthesiology and Operative Intensive Care, University Medicine Halle (Saale), 06112, Franzosenweg 1a, Halle (Saale), Germany

**Keywords:** Quantitative real‐time PCR, Droplet digital PCR, Endothelial cells, Gene expression, Vasoactive receptors

## Abstract

**Background:**

In the present study, two distinct PCR methods were used for the quantification of genetic material and their results were compared: real-time-PCR (qPCR; relative quantification) and droplet digital PCR (ddPCR; absolute quantification). The comparison of the qPCR and the ddPCR was based on a stimulation approach of microvascular endothelial cells in which the effect of a pro-inflammatory milieu on the expression of vasoactive receptors was investigated.

**Results:**

There was consistency in directions of effects for the majority of genes tested. With regard to the indicated dimension of the effects, the overall picture was more differentiated. It was striking that deviations were more pronounced if the measured values were on the extreme edges of the dynamic range of the test procedures.

**Conclusions:**

To obtain valid and reliable results, dilution series are recommended, which should be carried out initially. In case of ddPCR the number of copies per µl should be adjusted to the low three-digit range. With regard to qPCR it is essential that the stability and reliability of the reference genes used is guaranteed. Here, ddPCR offers the advantage that housekeeping genes are not required. Furthermore, an absolute quantification of the sample can be easily performed by means of ddPCR. Before using ddPCR, however, care should be taken to optimize the experimental conditions. Strict indications for this methodology should also be made with regard to economic and timing factors.

## Background

The polymerase chain reaction (PCR) is a chemical process that mimics the physiological process of replication in body cells. It enables the multiplication of deoxyribonucleic acid (DNA) sequences in high numbers *in vitro* and is used for amplification of target DNA segments. This *in vitro* amplification is applied in many different medical fields: in diagnosis, e.g. viral infections, for prenatal diagnostics, in the production of drugs using genetically modified microorganisms, and in forensic medicine to identify persons. In basic medical research, PCR is also frequently used to determine the extent of the expression change of a gene of interest in human cells under certain conditions.

One variant of PCR is the so-called quantitative real-time PCR (qPCR), in which a fluorescence signal allows quantification of the amplicon simultaneously with the synthesis. A SYBR Green-based qPCR was used in the present study. SYBR Green is a fluorescent dye, which binds to double-stranded DNA. As the amount of double-stranded DNA increases during PCR, the fluorescence increases as more and more SYBR Green is intercalated. In qPCR, the measured fluorescence signal of the target gene under consideration is related to that of a reference gene. This is based on the assumption of a stable expression of this control, also known as housekeeping gene, even under altered external conditions. Thus, the 2^−ΔΔCT^ calculation method can be used to make statements on how the expression of the target gene changes relatively [1]. In the present study the reference gene *18S* was used. The *18S* rRNA is an important component of all eukaryotic ribosomes and part of the small ribosomal subunit. *18S* has been used in numerous studies worldwide and is well established as a reference gene for qPCR [2].

The droplet digital PCR (ddPCR) is a method available since 2011 and allows an direct quantification of genetic material [3, 4]. In principle, the same reagents and process steps are used in the analysis as for qPCR. The special feature of the method is the division of the reaction batch prior to amplification in the PCR thermal cycler into approximately 20 to 25,000 reaction chambers in the form of nanodroplets. These droplets are generated by a water-oil emulsion and enclose the PCR reagents. After the amplification of the examined gene within the nanodroplets, the fluorescence signal of each individual compartment is registered and read out. The number of nanodroplets in which the target sequence was amplified is recorded. The readout differentiates between positive and negative nanodroplets. The respective amount of amplification per compartment is not considered. In order to include it in the analysis, the concentration of the target gene in the sample under investigation is determined using the Poisson distribution. The ddPCR represents an end-point measurement, which results in a present-or-absent digital format with clear thresholds between positive and negative droplet clusters. Therefore, quantification of nucleic acids can be performed without the need for external calibrators or endogenous controls and independent of reaction efficiency [5]. This results in a regime of absolute DNA quantification without the need for standard curves or reference genes [5]. In addition, the influence of PCR inhibitors and sample contamination is reduced to a minimum by endpoint measurement [6–8]. The detection of very rare target sequences is to be facilitated by compartmentalizing the overall PCR reaction, since here even single existing copies per nanodroplet can be detected [9]. Consequently, direct comparison between ddPCR and qPCR using the standard curve method revealed improved sensitivity of ddPCR [7] and excellent precision especially for low expressed target genes [8]. According to the manufacturer of the QX200™ Droplet Digital™ PCR System (Bio-Rad Laboratories) used for the present study, the linear dynamic measuring range of the ddPCR lies between 1 and 100,000 copies per 20 µl reaction [5].

The present study aimed to compare the two quantification methods qPCR and ddPCR. The model used was the endothelial expression of vasoactive receptors under inflammatory conditions. This model was selected in light of the main research focus of the investigators: sepsis-induced vasoplegia. In severe cases sepsis is characterized by an arterial hypotension along with vasodilatation, which accounts for death due to multi organ failure [10]. What is more, in septic patients the blood pressure response to vasoconstrictors is significantly reduced [10]. Previous animal model studies hint towards pro-inflammatory cytokines, which are increasingly present in the blood stream during sepsis, as causative factors by mediating a decline in the expression of vasoactive receptors [11–15]. To study this mechanism in more detail, valid methods to determine endothelial expression of vasoactive receptors are needed. The ddPCR seems promising for two reasons: (i) the method directly provides information not only on relative expression changes but also on the absolute expression level of the vasoactive receptors, (ii) by avoiding the need for reference genes, the risk of bias due to inflammation-related shifts in the expression of the housekeeping gene used is overcome. A prerequisite for the use of ddPCR is the knowledge of the reliability and validity of the data obtained by ddPCR compared to the standard qPCR procedure. There is, unfortunately, a lack of comparative investigations of both methods, which were performed independently (i.e. not performed or commissioned by the manufacturer) and using identical samples. This gap in knowledge was addressed by the present study.

## Results

### Indicated effect direction

**Fig. 1:**
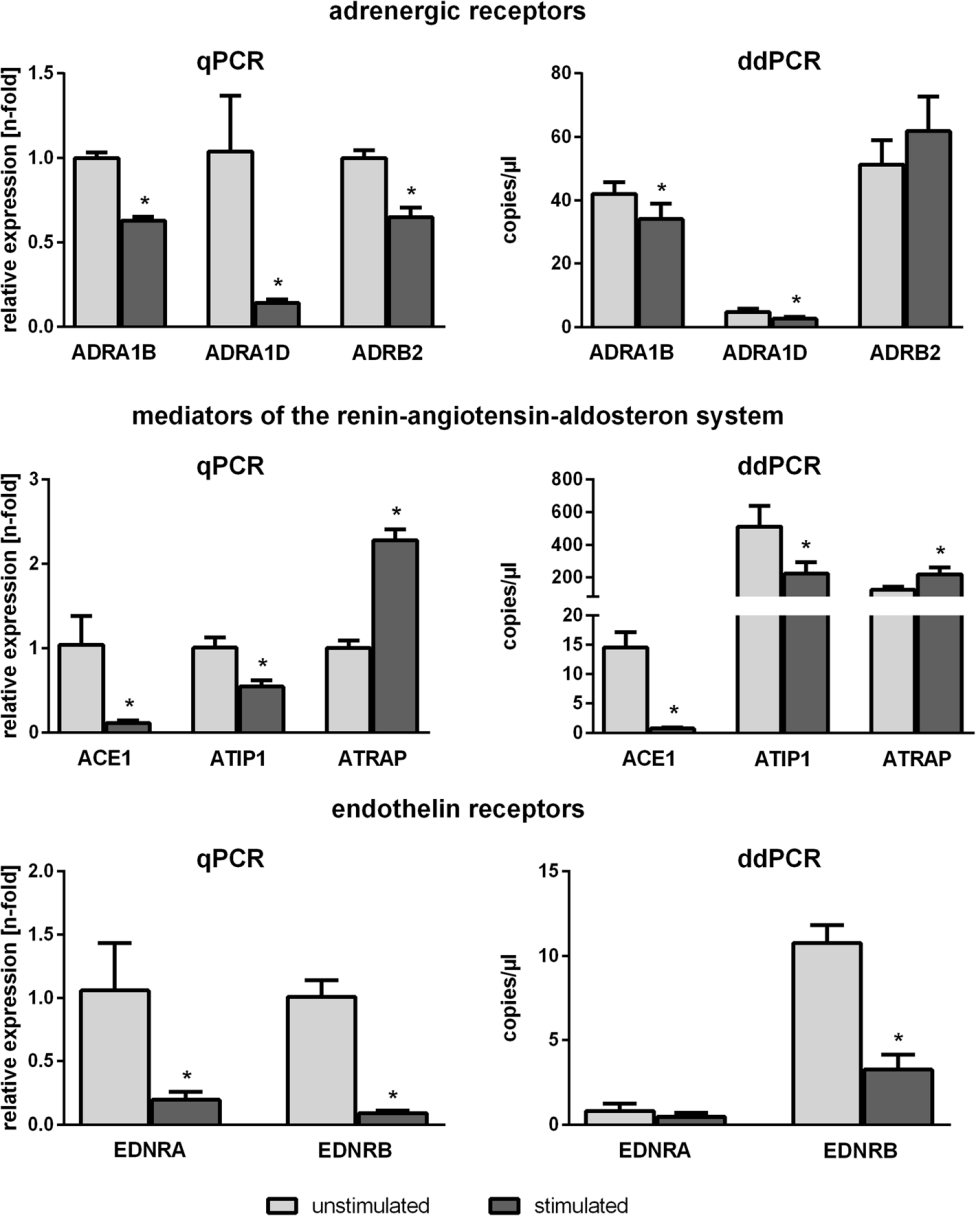
Effect direction indicated by qPCR and ddPCR. Human endothelial cells of the cell line TIME (ATCC number: CRL-4025) were stimulated for 24 h with TNF-α, IL-1β, and INF-γ in concentrations of 100 ng/ml each. The measured values are presented as mean values ± standard deviation. qPCR was performed in 5 independent experiments using 9 technical replicates each and ddPCR analysis was performed in 5 independent experiments using 3 technical replicates each. * symbolizes significant differences. *ACE1* = angiotensin converting enzyme 1, *ADRA1B* = alpha-adrenergic receptor type 1B, *ADRA1D* = alpha-adrenergic receptor type 1D, *ADRB2* = beta-adrenergic receptor type 2, *ATIP1* = angiotensin II receptor interacting protein 1, *ATRAP* = angiotensin II type 1 receptor associated protein, *EDNRA* = endothelin receptor type A, *EDNRB* = endothelin receptor type B

In 6 out of 8 target genes, there was agreement in the results of qPCR and ddPCR with regard to the effect direction. Thus, both qPCR and ddPCR indicated a reduction in gene expression for *ADRA1B*, *ADRA1D*, *ACE1*, *ATIP1*, and *EDNRB* as well as an increase in gene expression for *ATRAP* as a result of cell stimulation (Fig. 1). In the case of *EDNRA*, qPCR analysis revealed a significant reduction in gene expression whereas ddPCR showed only a corresponding trend. Conflicting data were obtained for *ADRB2*. While the qPCR-based analysis pointed toward a significant reduction in gene expression, the ddPCR-based analysis indicated a tendency towards increased gene expression (Fig. 1).

### Indicated effect size for genes found to match with regard to effect direction

**Fig. 2:**
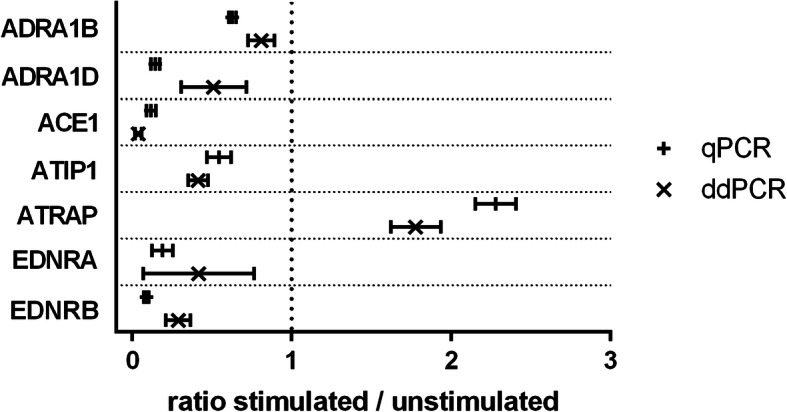
Effect size indicated by qPCR and ddPCR. Shown are the ratios of gene expression between stimulated and unstimulated cells, measured by qPCR or ddPCR. The value of 1, which corresponds to a lack of stimulation-induced gene expression change, is highlighted by a vertical dotted line. The measured values are shown as mean values ± standard deviation. qPCR was performed in 5 independent experiments using 9 technical replicates each and ddPCR analysis was performed in 5 independent experiments using 3 technical replicates each. *ACE1* = angiotensin converting enzyme 1, *ADRA1B* = alpha-adrenergic receptor type 1B, *ADRA1D* = alpha-adrenergic receptor type 1D, *ATIP1* = angiotensin II receptor interacting protein 1, *ATRAP* = angiotensin II type 1 receptor associated protein, *EDNRA* = endothelin receptor type A, *EDNRB* = endothelin receptor type B

The effect size was indicated by qPCR and ddPCR in a similar order of magnitude for 3 genes examined. This relates to the genes of interest *ADRA1B*, *ATIP1*, and *ATRAP*. With regard to *ADRA1B*, expression reduction by a factor of 0.6 was determined by qPCR and by a factor of 0.8 by ddPCR (Fig. 2). For *ATIP1*, a cytokine-induced reduction of mRNA synthesis to 0.5 times (qPCR) and 0.4 times (ddPCR) of unstimulated cells was observed (Fig. 2). Regarding *ATRAP*, qPCR showed a 2.3-fold increase and ddPCR a 1.8-fold increase of the initial expression rate (Fig. 2). For the target genes *ADRA1D*, *ACE1*, *EDNRA*, and *EDNRB*, however, larger deviations in the indicated effect size were found. The mRNA amount of *ADRA1D* was indicated to be reduced to 0.1 times (qPCR) versus 0.5 times (ddPCR) the value of the unstimulated control (Fig. 2). For *ACE1* a stimulation-induced expression reduction to 0.1 times (qPCR) versus 0.04 times (ddPCR) the value of unstimulated endothelial cells was found (Fig. 2). The expression of *EDNRA* was observed to be reduced to 0.2-fold (qPCR) versus 0.4-fold (ddPCR) of unstimulated control cells due to cytokine treatment (Fig. 2). And for *EDNRB* an expression reduction due to cytokine treatment of 0.09 times (qPCR) versus 0.3 times (ddPCR) the initial expression rate was indicated (Fig. 2).

There was no indication that one of the methods examined generally underestimated or overestimated the effect. However, a common feature of the genes was striking, which showed larger deviations in the indexed effect size: a low abundance at the limit of the dynamic range of the analytical procedures. On average, these genes were detected in an order of magnitude of 3.7 copies/µl or Ct 29.4 (*ADRA1D*), 7.7 copies/µl or Ct 28.0 (*ACE1*), 0.6 copies/µl or Ct 30.0 (*EDNRA*), and 7.0 copies/µl or Ct 25.7 (*EDNRB*). The data thus indicate that measured values at the limits of detectability are subject to a greater degree of uncertainty.

### Assessment of the abundance of the target gene

**Fig. 3:**
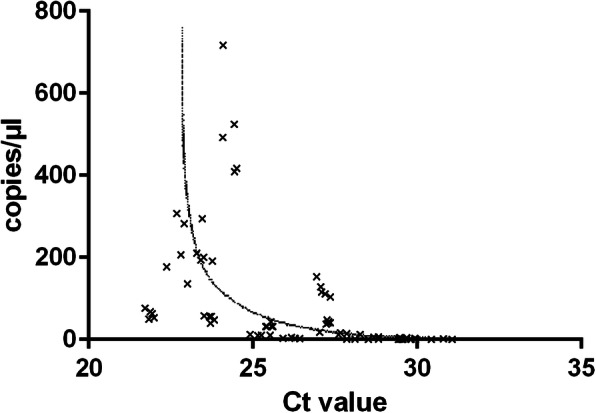
Abundance indicated by qPCR and ddPCR. Ct values measured by qPCR were plotted against the corresponding copies per µl measured for the identical samples by ddPCR. The resulting negative-exponential relationship is highlighted by a trend line. qPCR was performed in 5 independent experiments using 9 technical replicates each and ddPCR analysis was performed in 5 independent experiments using 3 technical replicates each

With an assumed efficiency of the PCR reaction of 100 %, a doubling of the target gene per cycle can be expected. Under ideal conditions, a reduction of the Ct by a value of 1 (e.g. from Ct 25 to Ct 24) would thus correspond to a doubling and a reduction of the Ct by a value of 2 (e.g. from Ct 25 to Ct 23) would correspond to a quadrupling of the amplicon. Consequently, when the number of copies measured by ddPCR is plotted against the Ct values measured by qPCR, a negative-exponential relationship is obtained (Fig. 3). However, Fig. 3 also shows that individual measured values may deviate significantly from the ideal course. It is, in any case, not feasible to derive the number of gene copies from the measured Ct value with sufficient accuracy. The Ct value obtained by qPCR just allows a rough estimate of the abundance of a target gene. To ascertain the concentration of a gene of interest in a sample in a reliable and straightforward way, it is mandatory to perform a ddPCR.

## Discussion

Absolute quantification using ddPCR has been commercially available since 2011 and is now increasingly used. However, even almost 10 years after the introduction of this method on the basis of the available studies, no clear recommendation for or against ddPCR can be given in relation to the long-term proven amplification method qPCR.

In the present study, the telomerase-immortalized endothelial cell line TIME (ATCC number: CRL-4025) was used as a model for microvascular endothelial cells. A major advantage of this cell line is its stability in expressing characteristic patterns of microvascular endothelial cells, such as CD31 or αVβ3-integrin, even after more than 25 population doublings [16]. The selection and dosage of the proinflammatory cytokine mixture used to stimulate endothelial cells paralleled the conditions found in inflammatory processes in the local cellular milieu or in the cerebrospinal fluid [17, 18].

To ensure high validity of the obtained results, all experimental steps were performed in a standardized manner. Furthermore, a high degree of comparability was ensured when performing qPCR and ddPCR. This includes (i) the use of identical reagents, primers, and materials at identical concentrations (with the exception of procedure-specific premixed supermix solutions), (ii) the use of identical negative and positive controls, and (iii) the harmonization of the programs of the thermal cyclers employed for qPCR and ddPCR. The risk of technically induced data bias is considered low, as the manufacturer’s instructions were strictly followed. Primers were optimized for high specificity and efficacy (i.e., short PCR product length, low melting temperature difference, no loop formation at annealing temperature) and validated by melting curve analysis as well as gel electrophoresis.

There was consistency in directions of effects for the majority of genes tested. With regard to the indicated dimension of the effects the overall picture was more differentiated. In some cases, there were clear disparities between qPCR and ddPCR. It was striking that deviations were more pronounced if the measured values were on the extreme edges of the dynamic range of the test procedures. According to the manufacturer, the dynamic range of ddPCR is 0.05 to 5,000 copies/µl [5]. However, this specification should be viewed rather critically. Test series published so far indicate a linear dynamic range of the ddPCR between 25 and 1,500 copies/µl with an ideal range of 100 to 1,000 copies/µl [19]. Also within this study, more uncertainties and higher scattering measures were noted if the results were below a limit of 10 copies/µl. The manufacturer’s statement of a dynamic range of 0.05 to 5,000 copies/µl thus rather indicates the possible range of detection and is not to be regarded as those limits within which valid results can be expected. The same applies to specifications for qPCR. The dynamic range of Ct values specified in scientific literature between 18 and 29 is greater than that of ddPCR [20]. However, measurement results obtained at or outside of these limits should be critically reviewed, as literature suggests that primer hybridization, dimer and loop formation, and the occurrence of unspecific secondary amplifications may occur more frequently [6, 7, 21]. To obtain valid and reliable results, dilution series are recommended, which should be carried out initially for qPCR and ddPCR. From our experience and in consultation with the manufacturer, we can suggest that for ddPCR, the number of copies per µl should be adjusted to the low three-digit range, ideally to about 100 copies/µl. We also recommend including several technical replicates, whereby the increase in validity and safety on the one hand and the additional time and expense on the other hand need to be carefully balanced.

With regard to qPCR, the stability and reliability of the reference genes used must be guaranteed. In meta-analyses of several tissue types it could be shown that in the presence of cytokine stimulation by messenger substances such as TNF-α the stability and reliability of reference genes such as β-actin (*ACTB*), glyceraldehyde-3-phosphate dehydrogenase (*GAPDH*,) or *18S* can be significantly influenced [22–24]. Indeed, in the present study, a weakness of the used reference gene *18S* was found, which was characterized by differences in the measured Ct values between unstimulated and stimulated samples by about 3 to 3.5. This significantly limits the validity of the data obtained by qPCR. It is reasonable to assume that some of the differences in the results obtained by qPCR and ddPCR are due to this instability of the housekeeping gene. Our data emphasize that the identification of appropriate reference genes for the experimental system under investigation is a mandatory prerequisite for obtaining valid results in qPCR. This necessity does not apply when ddPCR is used.

Compared to qPCR, ddPCR offers clear advantages. The most prominent one is the ability to easily quantify the amount of DNA copies present in a sample. Further advantages include the dispensability of external calibration using standard curves and the fact that housekeeping genes are not required. In addition, ddPCR is believed to inherit a higher resilience towards non-specific PCR inhibitors and background DNA, which allows a higher sensitivity and specificity to be achieved [6–8]. However, the increased resistance to PCR inhibitors should not be uncritically assumed. Studies have shown that the extent of this resilience of ddPCR is strongly dependent on the type and quantity of inhibitors present within the reaction compartments [25]. Clear superiority of ddPCR over qPCR was shown in areas where the detection of quite small copy numbers was necessary, e.g. of viral and cancerous mutation or in the detection of so-called copy number variants (CNV) [19, 26, 27].

The disadvantages of ddPCR are the increased time, workload and costs. The ddPCR requires an additional manual work step: the generation of nanodroplets. This additional step adds to the handling time and mandates the purchase of additional consumables. What is more, on the international market, these required materials can only be purchased from a few suppliers, creating a monopoly situation that limits the scientist’s room for price negotiation. In addition, the throughput within the process is reduced by the exclusive presence of 96-well plates and 8-well cartridges. The decision for one of the two analytical methods can therefore not be made on a global basis. Rather, the scientist must weigh up the above points in order to select the best method for the planned investigations.

## Conclusions

ddPCR is a suitable method for the absolute quantification of target gene sequences and could become increasingly important over the next few years. It offers some advantages over qPCR and is ahead of this method in its reproducibility for some experimental concepts and questions. Before using ddPCR, however, care should be taken to optimize the experimental conditions, for example by using dilution series to determine the optimum target gene concentrations within the reaction batch. Strict indications for this methodology should also be made with regard to economic and timing factors.

As a consequence of the data presented here, we have established a standard operating procedure (SOP) in our laboratory for performing ddPCR. A key point of this SOP is that for each target gene and experimental system, the optimal target gene concentration is routinely tested before the actual data collection is initiated. The ultimate goal is to pre-dilute the sample to such an extent that measured values are in the range of 100 copies/µl. This procedure has proven itself to lead to reliable and reproducible results independent of the performing experimenter. Under these conditions, the use of ddPCR can be recommended when absolute expression levels are to be detected or when the experimental system is characterized by fluctuations of classic reference genes.

## Materials and methods

### Aim, design, and setting of the study

In the present study, two distinct PCR methods were used for the quantification of genetic material and their results were compared: real-time-PCR (qPCR; relative quantification) and droplet digital PCR (ddPCR; absolute quantification). The comparison of the qPCR and the ddPCR was based on a stimulation approach of microvascular endothelial cells in which the effect of a pro-inflammatory milieu on the expression of vasoactive receptors was investigated.

### Materials

All chemicals and reagents were obtained from Sigma-Aldrich (Taufkirchen, Germany) unless noted otherwise. Cell culture flasks and plates were purchased from Greiner Bio-One (Frickenhausen, Germany). The human cell line TIME (CRL-4025; telomerase-immortalized dermal microvascular endothelial cell line) was purchased from ATCC (Manassas, USA).

### Cell culture and cytokine stimulation

Endothelial cells were cultured according to ATCC recommendations at 37 °C and 5 % CO_2_ in a humidified atmosphere. A basal microvascular endothelial growth medium enriched with 5 ng/ml VEGF, 5 ng/ml EGF, 5 ng/ml FGF, 15 ng/ml IGF-1, 10 mM L-glutamine, 0.75 U/ml heparin sulfate, 1 µg/ml hydrocortisone hemisuccinate, 50 µg/ml ascorbic acid, 5 % v/v FCS, and 12.5 µg/ml blasticidin (Provitro, Berlin, Germany) was used. For stimulation, the cells were first transferred to the supplement-free basal microvascular endothelial growth medium for 24 h. Then the stimulation was carried out by adding the cytokines IL-1β, TNF-α, and IFN-γ (all PeproTech, Hamburg, Germany) each in a concentration of 100 ng/ml over a period of 24 h.

### RNA isolation and cDNA synthesis

Total RNA was extracted utilizing the InviTrap Spin Cell RNA Mini kit (Stratec Biomedical AG, Birkenfeld, Germany). Gained RNA concentrations and the purity of the samples were checked using the NanoVue spectrophotometer (GE Healthcare, Solingen, Germany). To eliminate any DNA residues contained in the RNA samples, a DNAse digestion using DNAse I (2000 U/ml; New England Biolabs, Frankfurt (Main), Germany) was performed according to the manufacturer’s instructions. Complimentary DNA (cDNA) was synthesized using the qScript cDNA SuperMix from Quanta Biosciences (Gaithersburg, USA) according to the manufacturer’s instructions. A no-reverse transcriptase control was also prepared for each sample using a heat-inactivated qScript cDNA SuperMix.

### Primer design and establishment

The nucleotide database of the National Center for Biotechnology Information (NCBI; https://www.ncbi.nlm.nih.gov/nuccore/) was used to design the required primer pairs. In the case of multiple transcript variants, the one with the longest nucleotide sequence was chosen to take into account all other variants of the target gene. To ensure a high specificity of the primers, a PCR product length of 90–300 base pairs (bp) was chosen. The maximum melting temperature difference of the primers was minimized to ensure the most effective amplification of the desired sequence of the target gene by both the forward primer and the reverse primer. Subsequently, the “intron inclusion” function was activated. This function was used to ensure that forward and reverse primers were separated by at least one intron of the corresponding genomic DNA sequence. In the case of several transcript variants of the target gene, the range of the primers to be synthesized was additionally limited to that sequence which includes all transcript variants. The proposed primer variants for the desired target gene were then checked for temperature ranges of possible loop formation using the software GeneRunner (HelioGenetics, New Jersey, USA). Only those primer pairs were selected as sufficient where this temperature range and the annealing temperature of the primers were far apart. The primers were manufactured at Eurofins (Ebersberg, Germany). The determination of the optimal annealing temperatures of the selected primer pairs was done by gradient PCR. This was also used to exclude possible by-products during the amplification process. The primer sequences are shown in Table 1.

**Table 1:** Primer sequences and annealing temperatures (X) used for mRNA analysis

Target	Primer sequence (forward / reverse)	Product size	Annealing temperature [°C]
18S	GCATATGCTTGTCTCAAAGA / CCAAAGGAACCATAACTGAT	101 bp	55 °C
ACE1	AGCCCTCTCAGTGTCTACGC / CTCCTTGGTGATGCTTCCAT	187 bp	57 °C
ADRA1B	TCACGAGGACACCCTTAGCA / GGCTTCAGGGTGGAGAACAA	195 bp	57 °C
ADRA1D	TTCTTCTTTGTCCTGCCGCT / GAAGGCGCGCTTGAACTC	150 bp	57 °C
ADRB2	TGCTGACCAAGAATAAGGCCC / AATGGCATAGGCTTGGTTCGT	173 bp	61 °C
ATIP1	AAGCATTCGTCCAGCAGC / AGAGGTTTCATGCGCAGC	173 bp	55 °C
ATRAP	GAGCTCCTGGTCCACACTG / TAGAACGACCTCCCAGGCA	196 bp	65 °C
EDNRA	AACGAGATGGACAAGAACCGATGT / GACCGAGGTCATCAGACTTTTGGA	195 bp	62 °C
EDNRB	TGCTTGCTTCATCCCGTTCA / ACTTCCCGTCTCTGCTTTAGG	200 bp	59 °C

Cycling conditions: 95 °C, 3 min (one cycle) / 95 °C, 30 sec + X °C, 20 sec + 72 °C, 20 sec (45 cycles) / 72 °C, 5 min (one cycle) / 95 °C, 2 min (one cycle)

### Quantitative real‐time PCR (qPCR)

Gene expression was analyzed by means of a SYBR Green-based quantitative real-time PCR technology. qPCR was performed using the PerfeCTa SYBR Green FastMix (Quanta Biosciences, Gaithersburg, USA). Targets of interest were *ACE1*, *ADRA1B*, *ADRA1D*, *ADRB2*, *ATIP1*, *ATRAP*, *EDNRA*, and *EDNRB*. *18S* was used as housekeeping gene, as it is one of the most commonly applied reference genes in qPCR. Positive controls by means of human heart aorta total RNA (TaKaRa, Saint-Germain-en-Laye, France) as well as negative controls (i.e. no template control; no reverse transcriptase control) were performed in each run. Thermal cycling was carried out on the C1000 Touch™ Thermal Cycler (Bio-Rad Laboratories, Feldkirchen, Germany). The thermal cycling conditions for each target are shown in Table 1. Relative quantification was performed with the CFX-Manager™ software (Bio-Rad Laboratories, Feldkirchen, Germany) utilizing the 2^−ΔΔCT^ method.

### Droplet Digital PCR (ddPCR)

mRNA copy counts of *ACE1*, *ADRA1B*, *ADRA1D*, *ADRB2*, *ATIP1*, *ATRAP*, *EDNRA*, and *EDNRB* were determined by means of the housekeeping gene-independent droplet digital PCR technology (Bio-Rad Laboratories, Feldkirchen, Germany) following the manufacturer´s standard protocols and using ddPCR EvaGreen Supermix (Bio-Rad Laboratories, Feldkirchen, Germany). The thermal cycling conditions for each target are shown in Table 1. The ddPCR reaction was performed in a T100 Thermal Cycler (Bio-Rad Laboratories, Feldkirchen, Germany). Measurement of positive droplets per µl sample was performed on a QX200 ddPCR Droplet Reader (Bio-Rad Laboratories, Feldkirchen, Germany). Based on the droplet count and according to Poisson distribution, absolute nucleic acid copy count was calculated utilizing the software QuantaSoft (Bio-Rad Laboratories, Feldkirchen, Germany).

### Statistical analysis

qPCR was performed in 5 independent experiments using 9 technical replicates each and ddPCR analysis was performed in 5 independent experiments using 3 technical replicates each. In order to identify significant differences between means a paired parametric two-sided t-test was performed. The statistical analysis was carried out by means of the program GraphPad Prism 6 (GaphPad Software, La Jolla, USA). In all cases, *p* < 0.05 was assumed to indicate significant differences.

## Data Availability

The datasets used and/or analyzed during the current study are available from the corresponding author on reasonable request.
